# Differences in Inter-Rectus Distance and Abdominopelvic Function between Nulliparous, Primiparous and Multiparous Women

**DOI:** 10.3390/ijerph182312396

**Published:** 2021-11-25

**Authors:** Mercè Balasch-Bernat, Sofía Pérez-Alenda, Juan J. Carrasco, Begoña Valls-Donderis, Lirios Dueñas, Laura Fuentes-Aparicio

**Affiliations:** 1 Physiotherapy in Motion, Multi Speciality Research Group (PTinMOTION), Department of Physiotherapy, University of Valencia, 46010 Valencia, Spain; merce.balasch@uv.es (M.B.-B.); juan.j.carrasco@uv.es (J.J.C.); lirios.duenas@uv.es (L.D.); laura.fuentes@uv.es (L.F.-A.); 2 Department of Physical Education and Sports, University of Valencia, 46010 Valencia, Spain; vallsdon@alumni.uv.es

**Keywords:** inter-rectus distance, postpartum period, ultrasound imaging, abdominal muscles, parity

## Abstract

Widening of the inter-rectus distance (IRD) is highly prevalent among postpartum women and can lead to dysfunction of abdominopelvic muscles. The aim of this study was to evaluate the differences in IRD and abdominopelvic function between nulliparous, primiparous and multiparous women. A cross-sectional study was conducted on 75 women (25 nulliparous, 25 primiparous and 25 multiparous at 6 months postpartum). The participants underwent ultrasound assessment under three conditions (at rest, abdominal draw-in maneuver (ADIM) and curl-up) at two locations (2 cm above and 2 cm below the umbilicus). Furthermore, abdominopelvic muscle function was determined by prone, supine and side bridge tests. In all conditions and locations, the IRD were significantly higher (*p* < 0.05) in the primiparous and multiparous women than in the nulliparous. The multiparous women presented greater (*p* > 0.05) IRD at rest and during ADIM compared to the primiparous women. Regarding abdominopelvic muscle function, differences were only significant (*p* < 0.05) between the nulliparous with primiparous women in prone and supine conditions. These findings suggest that parity influences IRD: women at 6 months postpartum present greater IRD compared to nulliparous women; multiparous women present greater IRD at rest and during the activation of deep abdominal muscles than primiparous women; and primiparous women exhibit worse abdominopelvic muscle function than nulliparous women.

## 1. Introduction

Inter-rectus distance (IRD) refers to the separation between the two bellies of the rectus abdominis muscle along the linea alba [1,2]. The abnormal widening of the IRD is known as diastasis of the rectus abdominis (DRA). This condition can occur in both men and women; however, it is more prevalent in pregnant women during the third trimester as well as in postpartum [1,3]. Studies have found that DRA may affect between 30% and 70% of pregnant women [1], with a reported prevalence of 33.1%, 60.0%, 45.4%, and 32.6%, at gestation week 21, 6 weeks, 6 months, and 12 months postpartum, respectively [4]. During pregnancy, the increase in the dimensions of the uterus provokes an elongation of the rectus abdominis muscle, which may separate as an adaptive structural change [5]. Moreover, hormonal influence on the connective tissue increases this elongation [6]. This can lead to the dysfunction of the abdominopelvic muscles, including the rectus abdominis, the external and internal obliques, and the transversus abdominis [7]. Although these changes tend to decrease throughout the postpartum, several studies have shown that DRA can remain even until 6 months and 1 year postpartum [4,8].

The presence and severity of DRA is based on two important aspects: (1) the abdominal values of the IRD and (2) the alteration of the linea alba’s tensile properties and its ability to transfer the force [9,10]. In addition, there are several factors, such as surgeries and age, which can influence the presence of DRA. According to a recent study [11], abdominal surgeries in general and caesarean section in particular have a clear effect on DRA development, by altering abdominal fascial tissue and abdominal muscle function. In regard to age, current research [12,13] confirms its influence on DRA, indicating higher DRA prevalence amongst older women. It seems that the reduction of collagen levels due to aging alters connective tissue strength and elasticity, which can lead to DRA [14]. Thus, caesarean section and age have been considered as risk factors for DRA.

Regarding DRA effects, scientific research confirmed heterogeneous conclusions. On the one hand, a recent systematic review [15] found that there was no correlation between DRA and low back pain. On the other hand, DRA has been linked to pelvic floor dysfunction [12,13], pelvic and low back pain [16,17], and as lumbopelvic stability [17,18]. This can be explained by the synergistic functionality that exists between the pelvic floor, the erector spinae, and the abdominal muscles [19]. Furthermore, according to a recent study [20], women with DRA may present a lower quality of abdominal muscle contraction compared to controls. This fact may negatively influence pelvic floor muscle contraction, which registered lower contraction values. Besides, other studies [13,19] have observed that women with urinary incontinence show an alteration of the synergy between the pelvic floor muscles and abdominal muscles. Moreover, DRA exerts negative effects on quality of life and functionality [15]. For these reasons, it is considered important to assess and monitor IRD width over time, particularly when the patient presents with the aforementioned dysfunctions.

Several methods are used for the assessment of the IRD, such as measurement with callipers, tape measure, and the traditional ‘finger width‘ method [21]. Some authors claim that the ‘finger width’ method is ‘unreliable’ [22,23], with some research being more in favour of diagnosis through imaging tools such as ultrasound, CT, or MRI, which is considered the ‘gold standard’ for IRD measurement [18,23,24]. Ultrasound imaging, which has been used as a tool to determine the severity and impact of DRA, has been demonstrated to be a precise and reliable method for the assessment of IRD [25]. Data normality tests for IRD in postpartum women is still lacking and depends on the use of different localization references along the line alba [15]. Despite this, a widening greater than 2.2–2.3 cm identified by ultrasound measurements has been considered a clinically important DRA and has been linked to adverse effects [8,26].

To date, several authors [9,27,28] have studied IRD behaviour in postpartum women when compared with nulliparous women. Only Rett et al. [29,30] have specifically examined the differences in IRD between primiparous and multiparous. However, in their study, the IRD measures were performed using fingerbreadths and callipers. To our knowledge, there are no investigations comparing IRD and abdominopelvic function between nulliparous, primiparous, and multiparous women. The aim of this study was, therefore, to evaluate the differences in IRD by ultrasound imaging and abdominopelvic function between primiparous and multiparous women at 6 months postpartum, and nulliparous women. We hypothesized that both IRD and abdominopelvic function could be influenced by parity; women who have had more deliveries are more likely to present with greater IRD and poor abdominopelvic function.

## 2. Materials and Methods

### 2.1. Study Design

This was a cross-sectional observational study that conformed to the Declaration of Helsinki [31] and was approved by the University Research Ethics Committee (No. H1542709432243). All the participants were fully informed about the study’s purpose and procedures and provided written informed consent prior to participating. This article adheres to the STROBE guidelines [32].

### 2.2. Participants

A sample of nulliparous, primiparous, and multiparous women were recruited in Valencia, Spain, from November 2018 to January 2021, using flyers in obstetricians’ and other health care settings, as well as through prenatal education and prenatal fitness centers.

To be included in the study, the participants had to be aged between 23 and 43 years old (that is, around ten years older and younger than the mean age reference value obtained from the National Statistical Institute of Spain [33], which is 33 years old, considering both primiparous and multiparous women). Postpartum women who had had their last (vaginal) delivery 6 months before the study were included. Nulliparous women were recruited by convenience sample (i.e., university employees, public buildings around the university, through word-of-mouth advertising) to serve as a comparison group. This group was age-matched, taking the ages of postpartum women as the reference.

Women were excluded if they had a history of spinal, abdominal, and urogynecologic surgery, including caesarean birth or, any neuromuscular disease.

An a priori power analysis was performed using G* Power version 3.1.9.4 software (Heinrich-Heine-Universität, Düsseldorf, Germany) to calculate the sample size. With the study design, assuming a medium/large effect size f = 0.40 (d = 0.80), α = 0.05, and β = 0.15, a minimum sample size of 72 subjects was required.

### 2.3. Procedure

All the women attended a single assessment session. Two physical therapists with 10 years of clinical experience in women’s health (L.F.-A) and in rehabilitative ultrasound imaging (S.P.-A.) performed all the measurements. Firstly, L.F.-A. explained the procedure in detail to the participants. The same researcher performed an exhaustive anamnesis, in which all the women provided information about the following sociodemographic and clinical data: age, birth, baby weight, weight gain during pregnancy, soft tissue damage, profession, exercise before and during pregnancy, and routine. Weight, height, and waist circumference were measured using standard procedures. Profession was categorized according to the Daily Physical by Occupational Classification of the National Health and Nutrition Examination Survey [34]. Current exercise was classified based on the categories of exercise intensity described by Norton et al. (2010) [35] and the compendium of physical activities [36]. Moreover, the subjects were asked to mark their abdominal and low back pain intensity during the last 24 h by means of a Visual Analogue Scale (VAS, 0-100) [37], consisting of a line, 100 mm in length, anchored by word descriptors at each end (i.e., no pain and very severe pain). The participants were instructed to mark on the line the point that they felt best represented their perception of their current state. The score was determined by measuring in millimeters from the left-hand end of the line to the point that the patient marked, ranging from 0 = no pain to 100 = worst pain imaginable [38]. Next, all the women underwent an ultrasound imaging assessment led by S.P.-A. Details regarding the ultrasound assessment are described below. Lastly, abdominopelvic muscle function was determined by S.P.-A. using the prone bridge, supine bridge, and side bridge test.

### 2.4. Outcome Measures

#### 2.4.1. IRD Measurement

The IRD was assessed by ultrasound imaging. A SAMSUNG HS30 device (SAMSUNG MEDISON CO., LTD, Gangwon-do, Korea) interfaced with a two-dimensional, high-frequency linear transducer (LN 5-12) was used in B-mode for all the imaging.

The ultrasound transducer was placed transversely along the linea alba in order to register IRD at two locations: 2 cm above the superior border of the umbilicus and 2 cm bellow the inferior border of the umbilicus [23]. Good intratester and test-retest reliability of IRD measurement at these levels has been demonstrated [23]. The location of the transducer for each measurement was marked on the skin. The IRD was determined by measuring the length of a straight line connecting the medial edges of the bilateral rectus abdominis heads.

**Figure 1:**
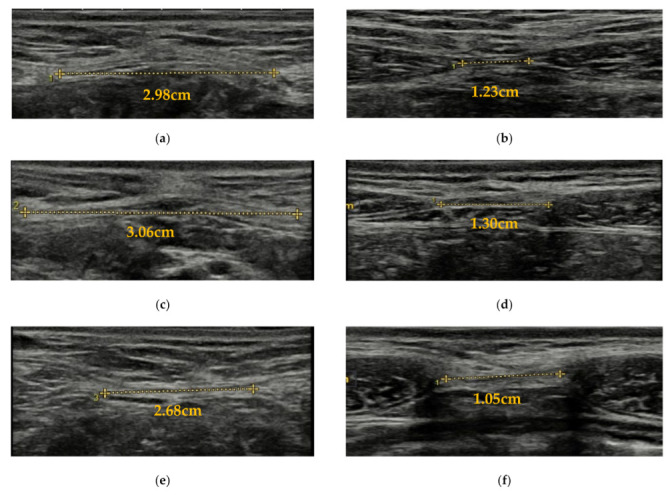
IRD measurement through ultrasound imaging assessment in the different conditions and locations. (**a**) IRD measurement at 2 cm above the umbilicus at rest; (**b**) IRD measurement at 2 cm below the umbilicus at rest; (**c**) IRD measurement at 2 cm above the umbilicus during ADIM; (**d**) IRD measurement at 2 cm below the umbilicus during ADIM; (**e**) IRD measurement at 2 cm above the umbilicus during curl-up; (**f**) IRD measurement at 2 cm below the umbilicus during curl-up. Abbreviations: IRD, inter-rectus distance; ADIM, abdominal draw-in maneuver.

Three still images were obtained at the two locations under three different conditions: resting in the supine position with a slight knee and hip flexion maintained by placing a pillow under the knees and with arms along the body, abdominal draw-in maneuver (ADIM) and abdominal curl-up (Figure 1). For the ADIM, participants were asked to first inhale and, after exhaling, to draw their belly button in towards the spine in order to activate the deep abdominal muscles. Each contraction was held for 3 s with 10 s rest between each contraction [23]. For the abdominal curl-up, the participants were instructed to lift their head and shoulder until the lower angle of the scapula lost contact with the table in order to activate the superficial abdominal muscles. They maintained this position until they were told to return to the resting position. The mean of the three images at each location under each condition was used for further analysis. In case the IRD was too large to be visualized through standard imaging, the panoramic mode was used so that the different images were sequentially joined to obtain the final image.

#### 2.4.2. Abdominopelvic Muscle Function

**Figure 2:**
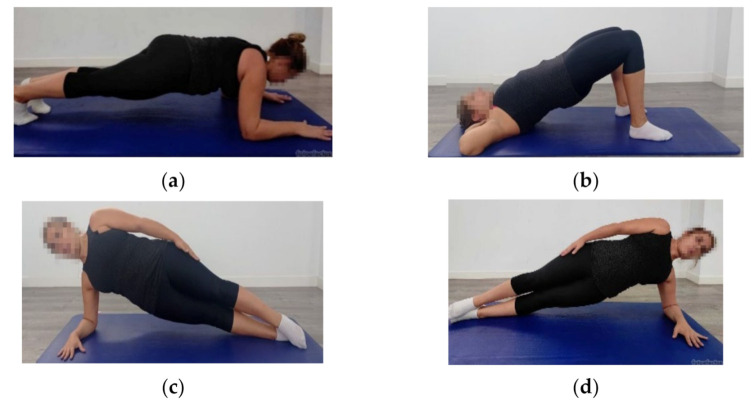
Abdominopelvic muscle function testing through prone bridge test (**a**), supine bridge test (**b**), and right and left side bridge test (**c**,**d**)**.**

Abdominopelvic muscle function was assessed by prone and supine bridge test and side bridge test (right and left) (Figure 2).

For the prone bridge test (Figure 2a), the women were positioned in the prone position, supported on their elbows at 90° flexion and shoulder-width apart, with their feet close but not touching. Next, the participants raised their pelvis from the floor, and the researcher ensured that their only supports were their forearms and toes, with the shoulders, pelvis, and ankles remaining aligned [39].

The supine bridge test (Figure 2b) consisted of subjects in the crook lying position, placing their hands by their ears. Next, the participants raised their pelvis, while maintaining their shoulders, hips, and knees aligned. In case the participants surpassed 2 min, their dominant leg was extended at the knee, remaining with a single point of support [39].

For the side bridge test (Figure 2c,d), the participants were positioned lying on their sides with their legs extended. The top foot was placed on the floor in front of the lower foot.

The subjects raised their pelvis from the floor, while maintaining their body alignment by supporting themselves only on one forearm and their feet. Their contralateral arm was placed across the chest with the hand on the opposite shoulder. The test was then repeated on the other side.

All the tests were performed on a mat. During all the tests, the subjects were reminded to maintain the position for as long as possible [40]. Each position was maintained until general, lumbar or abdominal fatigue, or until pain prevented the correct position. The time that the participants were able to maintain each posture was collected and was measured in seconds.

### 2.5. Data Analyses

The statistical analyses were performed using the IBM SPSS Statistics software for Windows (Version 26.0; IBM Corp, Armonk, NY, USA). The Shapiro–Wilk test was used to evaluate the normality of the data. The results are shown as mean (SD) or frequencies, as appropriate. The subjects’ demographic variables were compared using one-factor analysis of variance (ANOVA), Kruskall Wallis or chi-square (χ^2^).

To determine significant differences among the groups (nulliparous, primiparous, and multiparous), the conditions (rest, ADIM and curl-up) and locations (2 cm above and 2 cm below the umbilicus) in the IRD variable, three-factor ANOVA with repeated measures in factor condition and location were used. Differences among the groups in abdominopelvic muscle function were analysed using one-factor ANOVA. When the ANOVA models indicated significant differences in the main effects, Bonferroni correction was applied to avoid type I error in the multiple comparisons. The effect size was interpreted as small (d = 0.2; η_p_^2^ = 0.01), medium (d = 0.5; η_p_^2^ = 0.06), and large (d > 0.8; η_p_^2^ > 0.14). Statistical significance was set at *p* < 0.05.

## 3. Results

**Figure 3:**
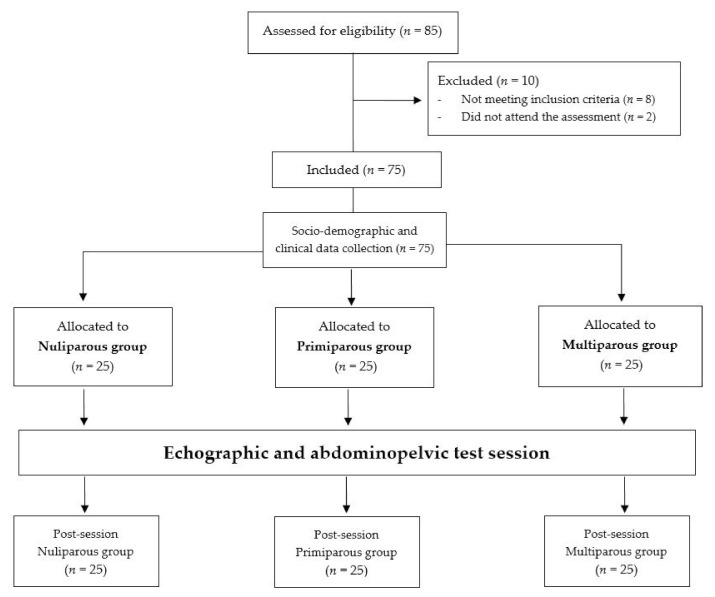
Flow diagram of participants.

The final sample of this study consisted of 75 women (25 nulliparous, 25 primiparous, and 25 multiparous), with a mean age of 34.15 ± 3.84 years. All the included subjects completed socio-demographic data collection as well as ultrasound assessment and abdominopelvic test sessions (Figure 3).

The participant’s demographic and clinical characteristics are presented in Table 1. The study groups did not show significant differences (*p* > 0.05) in age, weight, body mass index (BMI), height, waist circumference, profession physical activity classification, or in abdominal and low back pain. However, it was observed that the number of participants who performed physical exercise were significantly higher (*p* = 0.001) in the nulliparous than in the primiparous and multiparous groups. Furthermore, the nulliparous women registered higher exercise intensity (*p* = 0.007) than the multiparous women. Regarding the postpartum variables, both the primiparous and the multiparous women presented similar values, except for the rates of exercise during pregnancy. In this comparison, the primiparous group showed a significantly greater (*p* = 0.01) number of participants performing exercise than the multiparous group.

Table 2 summarizes the means (SD) of the outcomes and the results of the multiple comparisons. The ANOVA models showed that the IRD in the three conditions (rest, ADIM, and curl-up) and at the two locations (2 cm above and 2 cm below the umbilicus) were significantly higher (*p* < 0.05) in the primiparous and multiparous women than in nulliparous women. In all cases, the effect size was large. With regards to the comparison between the postpartum women, the multiparous women showed higher IRD than the primiparous women, with significant differences (*p* < 0.05) in the rest condition (only 2 cm above the umbilicus) and in the ADIM condition (at both locations).

Regarding the outcomes related to abdominopelvic muscle function, the nulliparous women showed better time values (i.e., higher scores) than the primiparous and multiparous women in the four conditions (prone, supine, and left and right sides). However, differences were only significant (*p* < 0.05) in the comparison between the nulliparous and primiparous women in prone and supine conditions. Finally, no significant differences were found in the time values between the primiparous and multiparous women.

## 4. Discussion

To the best of our knowledge, this is the first study investigating the differences in IRD assessed by ultrasound and abdominopelvic muscle function between primiparous and multiparous women at 6 months postpartum and nulliparous women. 

### 4.1. IRD Measurement

The results of this study reveal that both primiparous and multiparous women 6 months after giving birth showed higher IRD values when compared to nulliparous regardless of the condition and location of the measurement, confirming our hypothesis. Based on our results, at 6 months postpartum, the women presented greater IRD along any location of the linea alba not only in a resting position but also when activating deep (ADIM) or superficial (curl-up) abdominal muscles, compared to the nulliparous women. No studies have previously investigated IRD values comparing between nulliparous, primiparous and multiparous women. More specifically, the primiparous and multiparous women participating in our study showed an IRD at rest of 1.99 and 2.60 cm at 2 cm above the umbilicus, and 1.32 and 1.43 cm at 2 cm below, respectively. Previous studies have also reported IRD values at rest but considering women with heterogeneous parity (i.e., nulliparous and multiparous in the same group) and measurement locations along the linea alba. For instance, Liaw et al. [26], in a sample consisting of both primiparous and multiparous women at 6 months postpartum, reported lower IRD values compared to our results (1.80 at 2.5 cm above the umbilicus and 1.16 cm at 2.5 cm below the umbilicus). However, Mota et al. [28], in a sample including only primiparous participants, obtained higher IRD values (2.24 and 1.53 cm at the same assessment points as in the current study). Our study is unique in the assessment of the IRD in several conditions related to the activation of the abdominal muscles (rest, ADIM and curl-up) to better identify, in an objective way, the differences in IRD between nulliparous, primiparous, and multiparous women. In this sense, intra-group analysis showed that both primiparous and multiparous women presented statistically significant changes in IRD between the three conditions (rest–ADIM, rest–curl-up, ADIM–curl-up). This is in accordance with other authors [41], who observed IRD narrows during a curl-up in postpartum women. The only non-significant comparison was rest–curl-up at 2 cm below in both primiparous and multiparous women. This could be explained by the fact that the supraumbilical region in postpartum women has been demonstrated to have greater IRD values [29,42]. Moreover, we found that the nulliparous women presented statistically significant differences in IRD only between ADIM–curl-up at 2 cm above the umbilicus, showing a narrowing of the IRD from ADIM to curl-up. This finding is supported by the study of Lee and Hodges [9], in which the authors explain the effect of abdominal muscle activation on IRD. It seems that the activation of the transversus abdominis during ADIM leads to a widening of the IRD, while the activation of the rectus abdominis during a curl-up leads to a reduction in the IRD [9,42].

All these differences in IRD behavior between postpartum and nulliparous women confirm the morphological and functional changes in abdominopelvic muscles that have been reported in postpartum women in comparison to nulliparous women, mainly related to the elongation and thickness decrease of the abdominal muscles [43].

Moreover, our findings suggest that multiparous women present higher IRD values compared to primiparous, in both at rest and ADIM. However, while these differences were observed at 2 cm both above and below the umbilicus during ADIM, they were only detected 2 cm above the umbilicus at rest. These results again be explained by the greater IRD values in the supraumbilical region in postpartum women [29].

### 4.2. Abdominopelvic Muscle Function

Regarding abdominopelvic muscle function, in our study, only the primiparous women demonstrated statistically significant differences when compared with the nulliparous women, showing worse abdominopelvic muscle function in the performance of the prone and supine bridge tests. These results are in line with those of other authors, who have confirmed changes in abdominopelvic function, such as lower strength and higher fatigability of both trunk and lumbopelvic stabilizing muscles in postpartum women, which could explain our findings [43,44,45]. Surprisingly, when comparing the multiparous and nulliparous women, no statistically significant differences were obtained. These could partly be due to current exercise frequency and the presence of abdominal and low back pain in our sample. On the one hand, considering all postpartum women currently performing exercise, although non-significant, the multiparous women showed a trend towards the performance of exercise with a higher frequency (almost double time) than the primiparous women. On the other hand, considering the postpartum women’s abdominal or low back pain, the multiparous women showed a trend towards lower pain intensity (VAS) compared to the primiparous women (see Table 1). Moreover, no differences in abdominopelvic function were observed between the primiparous and multiparous women, suggesting that the abdominopelvic function recovery in both groups was similar at 6 months postpartum. Again, this could be explained by the differences in exercise frequency between the primiparous and multiparous women, indicating that although the number of multiparous women performing exercise was lower (see Table 1) and despite their possible family burden due to childcare in comparison to primiparous, those reporting that they exercised probably did so as more consolidated habit and with higher adherence. This is in line with the guidelines of the American College of Obstetricians and Gynecologists [46], which confirm that more frequent and progressive exercise offers clear benefits for the recovery of abdominal muscle function.

Considering all the above-mentioned findings, it is important to highlight that, although the multiparous women showed higher IRD than the primiparous women in some conditions (at rest and during the activation of the deep abdominal muscles), no differences in abdominopelvic muscle function were detected between the primiparous and multiparous women. Thus, it seems that although IRD is influenced by parity, this does not necessary indicate a negative effect on the global function of the abdominopelvic region. This is in accordance with other studies [9,10] which emphasize that to achieve a clear diagnosis of DRA, it is decisive to consider not only the IRD but also the behavior of the linea alba, since it allows the transmission of tensile forces along the midline of the abdomen. In the clinical context, this may indicate the importance of assessing abdominopelvic function in addition to the measurement of IRD in the management of women with DRA. Furthermore, currently, it is common for women diagnosed with DRA to receive catastrophic or alarmist messages regarding their condition, sometimes leading to the avoidance of movement (known as kinesiophobia). Our findings, on the contrary, encourage clinicians to promote active strategies aimed at achieving adequate competence of the muscles of the abdominopelvic region.

### 4.3. Limitations

In this study, there are some methodological issues that should be considered. First, the main weakness of this review is the small sample size and the performance of physical activity. These two aspects may have limited the generalizability of our results in postpartum women. Second, no information regarding breastfeeding was collected, which could be a factor influencing the natural course of the IRD and the abdominopelvic function during the postpartum period. Moreover, although exercise habits were registered (frequency and intensity), this study did not consider the effect of exercise on IRD and abdominopelvic muscle function. In future studies, it could be interesting to investigate this point by comparing groups performing different exercise modalities and/or intensities. Furthermore, the women in our sample exhibited relatively low BMI. Additionally, since this was a cross-sectional study, only data at 6 months postpartum were collected. Further research in this topic, including additional follow-up measurements throughout postpartum, is warranted to investigate the natural course of the IRD and the abdominopelvic muscle function in the longer term. 

## 5. Conclusions

In conclusion, parity influences IRD at 6 months postpartum, as both primiparous and multiparous women present greater IRD at rest as well as during the activation of the abdominal muscles compared to nulliparous women. Moreover, multiparous women present greater IRD at rest and during the activation of the deep abdominal muscles compared to primiparous women. Nevertheless, in our sample, weaker abdominopelvic muscle function was only observed in primiparous compared to nulliparous women. These findings may contribute to the understanding of the natural evolution of the IRD in postpartum women.

## Figures and Tables

**Table 1:** Demographic and descriptive data.

	Total (*n* = 75)	Nulliparous (*n* = 25)	Primiparous (*n* = 25)	Multiparous (*n* = 25)	Differences among Groups
**Sample characteristics**					
Age (y)	34.15 (3.84)	33.24 (4.58)	34.16 (2.56)	35.04 (4.04)	F = 1.39; *p* = 0.26
Weight (kg)	62.73 (10.62)	62.60 (9.17)	65.08 (13.08)	60.60 (9.28)	H = 1.57; *p* = 0.36
Body mass index (kg/m^2^)	22.67 (3.36)	22.34 (2.93)	23.45 (4.18)	22.26 (2.98)	H = 0.71; *p* = 0.41
Height (cm)	166.16 (5.93)	167.32 (6.72)	166.08 (5.32)	165.08 (5.69)	F = 1.05; *p* = 0.41
Waist circumference (cm)	83.15 (9.77)	80.40 (9.91)	86.00 (10.08)	83.29 (8.89)	H = 1.80; *p* = 0.14
Profession physical activity classification, *n* (%)					
High	19 (28.4%)	8 (36.4%)	7 (28.0%)	4 (20.0%)	χ^2^ = 3.61; *p* = 0.48
Medium	14 (20.9%)	5 (22.7%)	3 (12.0%)	6 (30.0%)	
Low	34 (50.7%)	9 (40.9%)	15 (60.0%)	10 (50.0%)	
Current exercise (Yes/No), *n* (%)	45 (60%)/30 (40%)	22 (88%)/3 (12%)	14 (56%)/11 (44%)	9 (36%)/16 (64%)	χ^2^ = 13.36; *p* = 0.001 *
Exercise frequency (h/w)	13.03 (17.17)	18.10 (22.05)	5.50 (3.62)	10.66 (9.49)	H = 7.14; *p* = 0.028 **
Exercise intensity, *n* (%)					
Vigorous	12 (16.0%)	7 (28.0%)	4 (16.0%)	1 (4.0%)	χ^2^ = 17.42; *p* = 0.007 ***
Moderate	16 (21.3%)	8 (32.0%)	6 (24.0%)	2 (8.0%)	
Light	17 (22.7%)	7 (28.0%)	4 (16.0%)	6 (24.0%)	
Sedentary	30 (40.0%)	3 (12.0%)	11 (44.0%)	16 (64.0%)	
Abdominal pain last 24 h (Yes/No), *n* (%)	6 (8%)/69 (92%)	4 (16%)/21 (84%)	1 (4%)/24 (96%)	1 (4%)/24 (96%)	χ^2^ = 2.84; *p* = 0.24
VAS (0–100)	41.17 (19.70)	49.25 (19.21)	30.00 (0.00)	20.00 (0.00)	-
Low back pain last 24 h (Yes/No), *n* (%)	26 (34.7%)/49 (65.3%)	11 (44%)/14 (56%)	5 (20%)/20 (80%)	10 (40%)/15 (60%)	χ^2^ = 3.25; *p* = 0.20
VAS (0–100)	41.42 (23.18)	43.73 (22.92)	51.20 (25.49)	34.00 (22.36)	F = 1.01; *p* = 0.38
**Postpartum sample characteristics**					
Birth weight of baby (kg)	-	-	3.31 (0.33)	3.27 (0.34)	t = 0.42; *p* = 0.68
Weight gain during pregnancy (kg)	-	-	12.30 (3.68)	12.14 (4.10)	t = 0.15; *p* = 0.89
Soft tissue damage, *n* (%)					
Not altered	-	-	4 (16%)	10 (40%)	χ^2^ = 4.62; *p* = 0.20
Episiotomy	-	-	10 (40%)	6 (24%)	
Perineal tearing	-	-	10 (40%)	9 (36%)	
Both	-	-	1 (4%)	0 (0%)	
Exercise before pregnancy (Yes/No), *n* (%)	-	-	18 (72%)/7 (28%)	16 (64%)/9 (36%)	χ^2^ = 0.39; *p* = 0.54
Frequency exercise (h/w)	-	-	7.53 (5.88)	11.89 (23.43)	t = −0.77; *p* = 0.45
Exercise during pregnancy (Yes/No), *n* (%)	-	-	19 (76%)/6 (24%)	10 (40%)/15 (60%)	χ^2^ = 6.65; ***p* = 0.010**
Frequency exercise (h/w)	-	-	12.32 (15.84)	19.3 (23.9)	t = −0.85; *p* = 0.41

Values are mean (standard deviation) unless otherwise indicated. Groups are compared using ANOVA (F), Kruskall Wallis (H) or chi-square (χ^2^). Significant differences are highlighted in bold. * Differences between primiparous and nulliparous as well as between multiparous and nulliparous. ** Differences between primiparous and nulliparous. *** Differences between multiparous and nulliparous. Abbreviations: VAS, Visual Analogue Scale.

**Table 2:** Results of the multiple comparison analysis for the inter-rectus distance and abdominopelvic muscle function variables.

		Nulliparous (*n* = 25)	Primiparous (*n* = 25)	Multiparous (*n* = 25)	Primiparous vs Nulliparous *p* [95% CI]; *d*	Multiparous vs Nulliparous *p* [95% CI]; *d*	Primiparous vs Multiparous *p* [95% CI]; *d*
IRD (cm)							
REST	2 cm above	1.23 (0.46)	1.99 (0.64)	2.60 (0.79)	**0.001 [0.29 to 1.24]; 1.38**	**<0.001 [0.91 to 1.83]; 3.38**	**0.012 [−1.1 to −0.11]; 1.27**
	2 cm below	0.52 (0.33)	1.23 (0.55)	1.43 (0.63)	**<0.001 [1.09 to 0.33]; 1.56**	**<0.001[1.28 to 0.55]; 1.81**	0.61 [−0.60 to 0.19]; 0.35
ADIM	2 cm above	1.31 (0.42)	2.13 (0.59)	2.78 (0.67)	**<0.001 [0.41 to 1.24]; 1.39**	**<0.001 [1.06 to 1.87]; 2.60**	**0.002 [−1.08 to −0.21]; 1.04**
	2 cm below	0.59 (0.33)	1.40 (0.53)	1.83 (0.65)	**<0.001 [0.42 to 1.19]; 1.50**	**<0.001 [0.87 to 1.62]; 2.40**	**0.027 [−0.84 to −0.04]; 0.07**
CURL-UP	2 cm above	1.09 (0.51)	1.68 (0.49)	1.91 (0.59)	**0.002 [0.19 to 0.99]; 1.18**	**<0.001 [0.43 to 1.20]; 1.49**	0.53 [−0.64 to 0.19]; 0.42
	2 cm below	0.50 (0.28)	1.06 (0.51)	1.23 (0.55)	**<0.001 [0.23 to 0.90]; 1.38**	**<0.001 [0.41 to 1.06]; 1.70**	0.73 [−0.52 to 0.18]; 0.32
REST vs ADIM *p* [95% CI]; *d*							
2 cm above		0.05 [−0.17 to 0.01]; 0.2	**0.002 [−0.24 to −0.04]; 0.23**	**<0.001 [−0.28 to −0.09]; 0.39**			
2 cm below		0.37 [−0.19 to 0.04]; 2.81	**0.011 [−0.31 to −0.03]; 0.31**	**<0.001 [−0.53 to −0.27]; 0.62**			
REST vs CURL-UP *p* [95% CI]; *d*							
2 cm above		0.38 [−0.08 to 0.35]; 0.29	**0.008 [0.07 to 0.56]; 0.56**	**<0.001 [0.45 to 0.92]; 1.59**			
2 cm below		1.00 [−0.19 to 0.22]; 3.30	0.26 [−0.07 to 0.40]; 0.32	0.08 [−0.02 to 0.42]; 0.34			
ADIM vs CURL-UP *p* [95% CI]; *d*							
2 cm above		**0.026 [0.02 to 0.42]; 0.52**	**<0.001 [0.23 to 0.69]; 0.85**	**<0.001 [0.66 to 1.09]; 1.39**			
2 cm below		0.75 [−0.10 to 0.29]; 0.29	**0.002 [0.11 to 0.56]; 0.65**	**<0.001 [0.39 to 0.82]; 1.00**			
Abdominopelvic muscle function (s)							
PRONE		21.05 (20.87)	11.21 (4.17)	12.60 (3.40)	**0.028** **[0.79 to 18.87]; 0.65**	0.08 [−0.59 to 17.48]; 0.57	1.00 [−10.51 to 7.75]; 0.37
SUPINE		43.95 (34.35)	25.64 (12.34)	27.97 (11.66)	**0.020** **[2.29 to 34.32]; 0.71**	0.05 [−0.04 to 31.98]; 0.62	1.00 [−18.68 to 14.00]; 0.02
LEFT		14.21 (15.78)	8.61 (4.00)	8.21 (3.03)	0.16 [−1.35 to 12.55]; 0.49	0.11[−0.94 to 12.96]; 0.53	1.00 [−6.68 to 7.50]; 0.01
RIGHT		14.07 (15.87)	7.95 (2.67)	9.63 (3.96)	0.10 [−0.84 to 13.08]; 0.54	0.37 [−2.52 to 11.40]; 0.38	1.00 [−8.78 to 5.42]; 0.50

Values are mean (standard deviation). Significant differences are highlighted in bold. Abbreviations: IRD, inter-rectus distance; ADIM, abdominal draw-in maneuver.

## Data Availability

The data presented in this study are available from the corresponding author upon reasonable request.
